# A study of clinical manifestations and associated factors in photosensitive patients with systemic lupus erythematosus

**DOI:** 10.1186/s40001-025-03795-7

**Published:** 2026-01-06

**Authors:** Jia-huan He, Zhi-qi Song, Hui-Li, Yi-jing Kang

**Affiliations:** 1 The Department of Dermatology, The Second Affiliated Hospital Zhejiang University School of Medicine, Hangzhou, 310009 Zhejiang China; 2 The Department of Dermatology, The First Affiliated Hospital of Dalian Medical University, Dalian, 116000 Liaoning China; 3 The Department of Pediatric, Taian 88 Hospital of China Rongtong Medical Healthcare Group Co. Ltd, Taian, 271000 Shandong China; 4 The Department of Nephrology, Taian 88 Hospital of China Rongtong Medical Healthcare Group Co. Ltd, Taian, 271000 Shandong China

**Keywords:** Systemic lupus erythematosus, Photosensitivity, Clinical feature, Associated factor, Early diagnosis

## Abstract

**Objective:**

Photosensitivity (PSN) is a common initial symptom of systemic lupus erythematosus (SLE). This study compared the clinical characteristics of PSN-SLE and non-PSN-SLE patients, analyzed their phenotypic differences and PSN-associated factors, and explored the diagnostic value of PSN for early SLE identification.

**Methods:**

A retrospective analysis was conducted on clinical data of SLE patients hospitalized at Taian 88 Hospital of China Rongtong Medical Healthcare Group Co. Ltd between October 2016 and October 2024. Patients were divided into photosensitive and non-photosensitive groups based on physician-confirmed PSN (defined as pathological skin reactions to ultraviolet radiation documented by at least two independent clinicians). Epidemiological, clinical, and immunological differences were compared, and PSN-associated factors were analyzed using SPSS 24.0. Normality tests (Shapiro–Wilk test) were performed for continuous variables; parametric tests (*t*-test) were used for normally distributed data, and non-parametric tests (Mann–Whitney *U*-test) for non-normally distributed data.

**Results:**

The overall PSN incidence in SLE patients was 36.59% (101/276). PSN was significantly more prevalent in female patients (95.05% vs. 86.86%, *P* < 0.05) and patients aged < 40 years and 40–60 years (79.21% vs. 60.57%, *P* < 0.05). Median (interquartile range) age at diagnosis was 41.0 (29.0–54.0) years for the total cohort, 39.0 (28.0–52.0) years for the PSN group, and 44.0 (31.0–56.0) years for the non-PSN group. PSN-SLE patients had a higher initial diagnosis accuracy (55.45% vs. 35.43%, *P* < 0.05). Multivariate logistic regression identified rash (OR = 21.019, 95%CI 5.473–80.728), alopecia (OR = 5.940, 95%CI 1.658–21.281), arthritis (OR = 1.889, 95%CI 1.045–3.416), Raynaud syndrome (OR = 2.641, 95%CI 1.443–4.834), anti-Sm antibody (OR = 1.408, 95%CI 1.091–1.818), anti-SSA antibody (OR = 2.337, 95%CI 1.510–3.615), anti-SSB antibody (OR = 3.084, 95%CI 1.519–6.626), decreased C3 (OR = 14.308, 95%CI 3.526–58.061), decreased C4 (OR = 3.429, 95%CI 1.321–8.904), and RF positivity (OR = 10.640, 95%CI 2.173–52.093) as independent PSN-associated factors.

**Conclusion:**

PSN is more common in female and young-to-middle-aged SLE patients. It correlates with distinct clinical (rash, alopecia, arthritis, Raynaud syndrome) and immunological (anti-Sm/SSA/SSB antibodies, decreased C3/C4, RF positivity) features. Routine screening of these markers in PSN patients may facilitate early SLE diagnosis.

**Supplementary Information:**

The online version contains supplementary material available at 10.1186/s40001-025-03795-7.

## Introduction

Systemic lupus erythematosus (SLE) is a complex autoimmune disease characterized by high morbidity and mortality, with an incompletely understood pathogenesis [1]. In China, SLE exhibits a relatively high prevalence of approximately 30–70 cases per 100,000 individuals, with this incidence showing an upward trend [2, 3]. Long-term survival rates among SLE patients are notably low, with 25–30-year survival rates standing at merely 30%. The high frequency of organ involvement in Chinese SLE patients may be associated with insufficient public awareness of early disease symptoms, leading to missed optimal diagnostic and therapeutic windows [3]. Consequently, enhancing public vigilance toward SLE to enable early diagnosis and preventive management—thereby improving patient prognosis—represents an urgent clinical priority [4].

Photosensitivity (PSN), defined as pathological skin reactions to ultraviolet (UV) radiation, constitutes a common manifestation of SLE. UV is recognized as a major environmental contributor to SLE pathogenesis and may represent the predominant etiological factor in specific contexts [5–7]. PSN remains a cardinal criterion in both the 1982 Edition [8] and the 1997 Edition [5] of SLE classification criteria released by the American College of Rheumatology (ACR).

Published evidence indicates that patients may initially present with isolated clinical features or immunological abnormalities suggestive of SLE, yet insufficient to fulfill complete ACR classification criteria. With disease progression over time, approximately 10–55% of such individuals ultimately develop classifiable SLE [9–13]. Despite being a frequent initial symptom of SLE, PSN is commonly overlooked during early disease stages. Establishing routine screening protocols for SLE in PSN patients—using ACR criteria as reference—could identify those not meeting full classification standards but exhibiting prodromal SLE manifestations. Therefore, we conducted a retrospective analysis of clinical manifestations, auxiliary examinations, and immunological profiles in 276 hospitalized SLE patients. By comparing clinical differences between PSN-SLE and non-PSN-SLE patients and analyzing PSN-associated factors, this study aims to establish an evidence-based framework for implementing standardized screening and follow-up systems for SLE in PSN individuals.

## Materials and methods

### Subjects and content

#### Subjects and grouping

Subjects were hospitalized patients diagnosed with SLE at Taian 88 Hospital of China Rongtong Medical Healthcare Group Co. Ltd from October 2016 to October 2024. PSN was defined as pathological skin reactions (e.g., erythema, rash) induced by UV exposure, confirmed by at least two independent clinicians based on medical history and physical examination. Patients were divided into photosensitive (PSN-SLE) and non-photosensitive (non-PSN-SLE) groups.

#### Diagnostic criteria and systemic manifestation assessment basis

##### Diagnostic criteria

The 1997 ACR classification criteria for SLE [5] (detailed in Supplementary Material 1)

##### Inclusion criteria

(1) Definite SLE diagnosis per 1997 ACR criteria; (2) adults aged ≥ 18 years; (3) no severe systemic diseases (malignancies, severe cardio-cerebrovascular diseases) or other autoimmune diseases

##### Exclusion criteria

(1) severe systemic diseases or other autoimmune diseases; (2) incomplete clinical data; and (3) photosensitivity due to non-SLE causes (e.g., drug-induced reactions, familial photosensitivity disorders)

##### Ethical consideration

We received approval from the Medical Ethics Committee of the China Rongtong Medical & Healthcare Group Tai’an 88 Hospital for undertaking this study. This study was approved by the Medical Ethics Committee of Taian 88 Hospital of China Rongtong Medical Healthcare Group Co. Ltd (Ethical Approval ID: RTYL88-2023-099) and complies with the Declaration of Helsinki. All participants provided written informed consent and could withdraw voluntarily. The study was designed to be secure and fair to patients while minimizing the risk of harm to participants.

##### SLE disease activity

Disease activity was assessed using the Systemic Lupus Erythematosus Disease Activity Index 2000 (SLEDAI-2000) [14]. Specifically, the standardized SLEDAI-2000 scores of 276 patients were calculated to evaluate SLE disease activity. This allowed for a comparative analysis of clinical phenotypic differences between the photosensitive and non-photosensitive groups. Scores were calculated based on clinical and laboratory findings within the past 10 days; higher scores indicate greater disease activity. Detailed SLEDAI-2000 scoring criteria are provided in Supplementary Material 2.

##### Assessment criteria for organ damage

Organ damage was defined based on SLE-related clinical and laboratory abnormalities, referenced to the SLE International Collaborating Clinics (SLICC) Damage Index [11] (detailed in Supplementary Material 3).

### Research content

#### Clinical data collection

1. Demographics: Age, sex, ethnicity, weight, height, living environment, marital status, occupation (collected via electronic medical records and structured telephone follow-up; recall bias was minimized by using standardized questionnaires and cross-verifying with medical records).
2. Disease onset and treatment

First onset status, disease course, initial symptoms, clinical manifestations (sorted by specificity: photosensitivity, rash (malar/discoid/other SLE-specific), renal damage, arthritis, alopecia, hematological damage, oral ulcer, fever, CNS diseases, serositis, cardiac damage, Raynaud syndrome, Sjögren), and misdiagnosis history.

#### Key laboratory tests and auxiliary examinations

Laboratory tests: Blood/urine routines, 24-h urinary protein, autoantibodies (ANA, anti-dsDNA, anti-Sm, anti-SSA, anti-SSB, anti-RNP), immunoglobulin (IgG/IgA/IgM), complement (C3/C4), erythrocyte sedimentation rate (ESR), rheumatoid factor (RF), C-reactive protein (CRP). Auxiliary examinations: Cardiac/abdominal ultrasound, ECG, head CT/MRI, chest X-ray/CT, renal biopsy.

Auxiliary examinations: Cardiac/abdominal ultrasound, ECG, head CT/MRI, chest X-ray/CT, renal biopsy.

### Statistical analysis

SPSS 24.0 was used for analysis. Normality of continuous variables was tested by Shapiro–Wilk test. Normally distributed data were presented as mean ± standard deviation ${\overline{x}}$ ± s) and compared by t-test; non-normally distributed data were presented as median (interquartile range) and compared by Mann–Whitney U-test. Enumeration data were described as n (%) and compared by *χ*^2^ test or Fisher’s exact test. Multivariate logistic regression identified PSN-associated factors. Missing data (accounting for < 5% of total) were imputed by multiple imputation. *P* < 0.05 was statistically significant.

## Results

### General data

#### Age

Median (IQR) age at diagnosis was 41.0 (29.0–54.0) years for 276 SLE patients. Patients were stratified into < 40 years (*n* = 132, 47.8%), 40–60 years (*n* = 114, 41.3%), and > 60 years (*n* = 30, 10.9%). PSN incidence was highest in the < 40 years group (45.5%), followed by 40–60 years (31.6%) and > 60 years (13.3%) (*χ*^2^ = 12.846, *P* < 0.01) (Table 1).

**Table 1:** age at diagnosis of SLE patients

Age group	Total (*n* = 276)	PSN-SLE (*n* = 101)	Non-PSN-SLE (*n* = 175)	*χ*^2^	*P*
< 40 years	132 (47.8%)	59 (45.5%)	73 (54.5%)	12.846	0.002
40–60 years	114 (41.3%)	36 (31.6%)	78 (68.4%)		
> 60 years	30 (10.9%)	6 (13.3%)	24 (86.7%)		

SLE: systemic lupus erythematosus; PSN: photosensitivity; IQR: interquartile range

#### Sex

The male–female ratio was 1:9 (28 males, 248 females). PSN incidence was higher in females (95.05% vs. 86.86%, *χ*^2^ = 4.715, *P* = 0.030) (Table 2).

**Table 2:** Sex characteristics of SLE patients

Sex	Total (*n* = 276)	PSN-SLE (*n* = 101)	Non-PSN-SLE (*n* = 175)	*χ*^2^	*P*
Female	248 (89.86%)	96 (95.05%)	152 (86.86%)	4.715	0.030
Male	28 (10.14%)	5(4.95%)	23 (13.14%)		

SLE: systemic lupus erythematosus; PSN: photosensitivity

### Accuracy rate of initial diagnosis

Overall, 118 (42.75%) patients were correctly diagnosed initially. Misdiagnoses (*n* = 158) were sorted by frequency: infectious disease (47, 17.03%), skin disease (30, 10.87%), renal disease (19, 7.61%), arthritis (22, 7.97%), hematological disease (22, 7.97%), other connective tissue disease (11, 3.99%), and others (7, 2.53%). PSN-SLE patients had higher correct diagnosis rates (55.45% vs. 35.43%, *χ*^2^ = 10.484, *P* = 0.001) (Table 3).

**Table 3:** Initial diagnosis of SLE patients

Initial diagnosis	Total (*n* = 276)	PSN-SLE (*n* = 101)	Non-PSN-SLE (*n* = 175)	*χ*^2^	*P*
SLE	118 (42.75%)	56 (55.45%)	62 (35.43%)	10.484	0.001
Infectious disease	47 (17.03%)	12 (11.88%)	35 (20.00%)	2.988	0.084
Skin disease	30 (10.87%)	4 (3.96%)	26 (14.86%)	7.849	0.005
Renal disease	19 (6.88%)	2 (1.98%)	17 (9.71%)	5.976	0.015
Others	72 (26.09%)	27 (26.73%)	45 (25.71%)	0.032	0.858

SLE: systemic lupus erythematosus; PSN: photosensitivity

### Initial symptoms

Initial symptoms sorted by frequency: rash (77, 27.90%), fever (56, 20.29%), arthritis (48, 17.39%), renal damage (44, 15.94%), hematological damage (25, 9.06%), fatigue (14, 5.07%), Raynaud syndrome (6, 2.17%), others (6, 2.17%). PSN-SLE patients were more likely to present with rash (43.56% vs. 18.86%, *P* < 0.001) or Raynaud syndrome (4.95% vs. 0.57%, *P* = 0.016); non-PSN-SLE patients were more likely to present with renal damage (21.14% vs. 6.93%, *P* = 0.002) or hematological damage (12.57% vs. 2.97%, *P* = 0.007) (Table 4).

**Table 4:** Analysis of initial symptoms in 276 SLE patients

Photosensitive group					Non-photosensitive group			
Symptom	Number of cases	Percentage (%)	Number of cases	Percentage (%)	Number of cases	Percentage (%)	*χ*^2^	*P*
Rash	77	27.90	44	43.56	33	18.86	19.434	< 0.001
Arthritis	48	17.39	18	17.82	30	17.14	0.021	0.886
Fever	56	20.29	20	19.80	36	20.57	0.023	0.878
Renal damage	44	15.94	7	6.93	37	21.14	9.653	0.002
Hematological damage	25	9.06	3	2.97	22	12.57	7.166	0.007
Fatigue	14	5.07	2	1.98	12	6.86	3.163	0.075
Raynaud syndrome	6	2.17	5	4.95	1	0.57	5.775	0.016
Other	6	2.17	2	1.98	4	2.29	0.028	0.867
Total	276	100.00	101	100.00	175	100.00	–	–

SLE: systemic lupus erythematosus; PSN: photosensitivity

### Clinical manifestations for diagnosing SLE

Common clinical manifestations (sorted by frequency): rash (189, 68.48%), arthritis (172, 62.32%), hematological damage (148, 53.62%), alopecia (127, 46.01%), fever (113, 40.94%), myositis (110, 39.86%), renal damage (107, 38.77%). PSN-SLE patients had higher rates of rash (99.01% vs. 50.86%), alopecia (61.39% vs. 37.14%), arthritis (71.29% vs. 57.14%), and Raynaud syndrome (20.79% vs. 9.71%) (all *P* < 0.05) (Table 5).

**Table 5:** analysis of clinical manifestations for diagnosing SLE in SLE patients

Symptom	Number of cases	Percentage (%)	Photosensitive group		Non-photosensitive group		*χ*^2^	*P*
			Number of cases	Percentage (%)	Number of cases	Percentage (%)		
Rash	189	68.48	100	99.01	89	50.86	68.791	< 0.001
Alopecia	127	46.01	62	61.39	65	37.14	15.152	< 0.001
Oral ulcer	41	14.86	17	16.83	24	13.71	1.798	0.483
Sjogren	107	38.77	31	30.69	76	43.43	4.376	0.036
Myositis	110	39.86	35	34.65	75	42.86	1.798	0.180
Fever	113	40.94	31	30.69	82	46.86	6.920	0.009
Arthritis	172	62.32	72	71.29	100	57.14	5.456	0.020
Raynaud syndrome	38	13.77	21	20.79	17	9.71	6.619	0.010
Renal damage	107	38.77	38	37.62	69	39.43	0.088	0.767
Hematological damage	148	53.62	44	43.56	104	59.43	6.481	0.011
Neuropsychiatric damage	35	12.68	13	12.87	22	12.57	0.005	0.943
Serositis	58	21.01	17	16.83	41	23.43	1.679	0.195
Total	276	100.00	101	100.00	175	100.00	–	–

SLE: systemic lupus erythematosus; PSN: photosensitivity

### SLEDAI score

Median (IQR) SLEDAI score was 18.0 (12.0–25.0) (severe activity: 111, 40.22%; moderate: 79, 28.62%; mild: 81, 29.35%; inactive: 5, 1.81%). No significant difference was found between PSN-SLE and non-PSN-SLE groups (*P* > 0.05) (Table 6).

**Table 6:** SLEDAI score of SLE patients

SLEDAI score	Number of cases	Percentage (%)	Photosensitive group		Non-photosensitive group		*χ*^2^	*P*
			Number of cases	Percentage (%)	Number of cases	Percentage (%)		
Essentially no activity	5	1.81	2	1.98	3	1.71	0.025	0.873
Mild activity	81	29.35	30	29.70	51	29.14	0.010	0.922
Moderate activity	79	28.62	29	28.71	50	28.57	0.001	0.980
Severe activity	111	40.22	40	39.60	71	40.57	0.025	0.875
Total	276	100.00	101	100.00	175	100.00	–	–

SLE: systemic lupus erythematosus; PSN: photosensitivity; SLEDAI: Systemic Lupus Erythematosus Disease Activity Index; IQR: interquartile range

### Immunological indicators

#### Autoantibodies

PSN-SLE patients had higher positive rates of anti-Sm (40.59% vs. 28.00%), anti-SSA (72.28% vs. 56.57%), and anti-SSB (30.69% vs. 11.43%) (all *P* < 0.05) (Table 7).

**Table 7:** analysis of autoantibody in 276 SLE patients

Item	Number of cases	Percentage (%)	Photosensitive group		Non-photosensitive group		*χ*^2^	*P*
			Number of cases	Percentage (%)	Number of cases	Percentage (%)		
ANA positive	275	99.64	101	100	174	99.43	0.579	0.447
Anti-RNP antibody	93	33.70	41	40.59	52	29.71	3.393	0.065
Anti-dsDNA antibody	148	53.62	55	54.46	93	53.14	0.044	0.833
Anti-Sm antibody	90	32.61	41	40.59	49	28.00	4.622	0.032
Anti-SSA antibody	172	62.32	73	72.28	99	56.57	6.727	0.009
Anti-SSB antibody	51	18.48	31	30.69	20	11.43	15.777	< 0.001
Total	276	100.00	101	100.00	175	100.00	–	–

SLE: systemic lupus erythematosus; PSN: photosensitivity; ANA: antinuclear antibody; dsDNA: double-stranded DNA; Sm: Smith; SSA: Sjögren syndrome A; SSB: Sjögren syndrome B; RNP: ribonucleoprotein

#### Complement

PSN-SLE patients had higher rates of decreased C3 (81.19% vs. 69.14%) and C4 (58.42% vs. 43.43%) (all *P* < 0.05) (Table 8).

**Table 8:** analysis of abnormal immunoglobulin and complement in 276 SLE patients

Photosensitive group					Non-photosensitive group			
Item	Number of cases	Percentage (%)	Number of cases	Percentage (%)	Number of cases	Percentage (%)	*χ*^2^	*P*
Elevated IgG	158	57.25	62	61.39	96	54.86	1.115	0.291
Elevated IgA	47	17.03	19	18.81	28	16.00	0.358	0.549
Elevated IgM	48	17.39	14	13.86	34	19.43	1.382	0.240
Decreased C3	203	73.55	82	81.19	121	69.14	4.776	0.029
Decreased C4	135	48.91	59	58.42	76	43.43	5.172	0.023
Total	276	100.00	101	100.00	175	100.00	–	–

SLE: systemic lupus erythematosus; PSN: photosensitivity; IgG: immunoglobulin G; IgA: immunoglobulin A; IgM: immunoglobulin M; C3: complement 3; C4: complement 4

#### RF

PSN-SLE patients had higher RF positivity (30.69% vs. 13.71%, *P* = 0.001) (Table 9).

**Table 9:** Analysis of abnormal ESR, RF, and CRP in 276 SLE patients

Item	Number of cases	Percentage (%)	Photosensitive group		Non-photosensitive group		χ^2^	*P*
			Number of cases	Percentage (%)	Number of cases	Percentage (%)		
Increased ESR	162	58.70	55	54.46	107	61.14	1.181	0.277
RF positive	55	19.93	31	30.69	24	13.71	11.570	0.001
Elevated CRP	135	48.91	48	47.52	87	49.71	0.123	0.726
Total	276	100.00	101	100.00	175	100.00	–	–

SLE: systemic lupus erythematosus; PSN: photosensitivity; ESR: erythrocyte sedimentation rate; RF: rheumatoid factor; CRP: C-reactive protein

### Logistic regression analysis of photosensitivity associated factors

Independent PSN-associated factors included rash (OR = 21.019), alopecia (OR = 5.940), arthritis (OR = 1.889), Raynaud syndrome (OR = 2.641), anti-Sm antibody (OR = 1.408), anti-SSA antibody (OR = 2.337), anti-SSB antibody (OR = 3.084), decreased C3 (OR = 14.308), decreased C4 (OR = 3.429), and RF positivity (OR = 10.640) (all P < 0.05) (Table 10).

**Table 10:** Logistic regression analysis of photosensitivity influencing factors in SLE patients

Variable	B	SE	Walds *χ*^2^	*P*	OR	OR 95%CI	Correlation
Rash	3.045	0.687	19.675	< 0.001	21.019	5.473–80.728	Positive
Alopecia	1.782	0.651	7.487	0.006	5.940	1.658–21.281	Positive
Oral ulcer	0.006	0.513	0.000	0.991	1.006	0.368–2.751	None
Sjogren	− 2.868	0.833	11.841	0.001	0.057	0.011–0.291	Negative
Myositis	− 1.138	1.341	0.721	0.396	0.320	0.023–4.435	None
Fever	− 1.609	0.753	4.563	0.033	0.200	0.046–0.876	Negative
Arthritis	0.636	0.302	4.433	0.035	1.889	1.045–3.416	Positive
Raynaud syndrome	0.971	0.308	9.921	0.002	2.641	1.443–4.834	Positive
Renal damage	− 0.313	0.452	0.478	0.489	0.731	0.302–1.774	None
Hematological damage	− 0.827	0.317	6.807	0.009	0.438	0.235–0.814	Negative
Anti-RNP antibody	0.511	0.660	0.599	0.439	1.667	0.457–5.085	None
Anti-dsDNA antibody	0.406	0.549	0.546	0.460	1.500	0.511–4.402	None
Anti-Sm antibody	0.342	0.130	6.894	0.009	1.408	1.091–1.818	Positive
Anti-SSA antibody	0.849	0.223	14.528	< 0.001	2.337	1.510–3.615	Positive
Anti-SSB antibody	1.126	0.361	9.717	0.002	3.084	1.519–6.626	Positive
Elevated IgG	0.191	0.303	0.397	0.529	1.210	0.668–2.190	None
Elevated IgA	0.193	0.415	0.216	0.642	1.213	0.537–2.736	None
Elevated IgM	− 0.394	0.406	0.941	0.332	0.674	0.304–1.495	None
Decreased C3	2.661	0.715	13.862	< 0.001	14.308	3.526–58.061	Positive
Decreased C4	1.232	0.487	6.408	0.011	3.429	1.321–8.904	Positive
Increased ESR	− 0.294	0.173	2.870	0.090	0.745	0.531–1.047	None
RF positive	2.365	0.810	8.513	0.004	10.640	2.173–52.093	Positive
Elevated CRP	0.465	0.343	1.832	0.176	1.592	0.812–3.119	None
Constant	− 29.906	3403.342	< 0.001	0.993	–	–	–

SLE: systemic lupus erythematosus; PSN: photosensitivity; OR: odds ratio; CI: confidence interval; SE: standard error; dsDNA: double-stranded DNA; Sm: Smith; SSA: Sjögren syndrome A; SSB: Sjögren syndrome B; RNP: ribonucleoprotein; IgG: immunoglobulin G; IgA: immunoglobulin A; IgM: immunoglobulin M; C3: complement 3; C4: complement 4; ESR: erythrocyte sedimentation rate; RF: rheumatoid factor; CRP: C-reactive protein

## Discussion

SLE represents a complex, systemic, and potentially fatal autoimmune disorder characterized by diverse clinical manifestations with significant interindividual variability. The disease spectrum may evolve from mild presentations such as isolated PSN, skin rash, or Raynaud syndrome to progressive involvement of vital organs (e.g., renal damage). Early identification of suggestive clinical features—prior to fulfillment of SLE classification criteria—through recognition of prodromal symptoms and predictive biomarkers could facilitate timely detection of immune dysregulation. Implementing longitudinal surveillance in such cases would enable earlier diagnosis and intervention, potentially reducing severe complications and mortality. Our findings demonstrate lower misdiagnosis rates and higher initial diagnostic accuracy in PSN-SLE patients, suggesting PSN may serve as a critical entry point for SLE screening initiatives.

Our epidemiological analysis of 276 SLE patients identified PSN in 36.59% of cases. Heightening vigilance toward SLE among PSN individuals and enhancing clinician awareness of PSN patients with SLE predisposition hold significant implications for early disease management. UV avoidance strategies—including sun protection, protective clothing, and UV-blocking sunscreen—can effectively prevent SLE development in susceptible individuals and mitigate characteristic skin lesions in established patients [15–18].

PSN was more prevalent in females and younger patients (< 40 years), aligning with SLE’s epidemiological characteristics [19, 20]. This observation corroborates findings from a cohort of 11,934 SLE patients where PSN incidence was higher in females [20], consistent with our results. Concerning age characteristics, peak PSN occurrence has been reported in patients aged 15–49 years [21], consistent with our data showing a younger age distribution in PSN versus non-PSN-SLE patients. Females may be more susceptible due to sex hormone-mediated immune dysregulation [19], while younger individuals have higher UV exposure and more active immune responses [21]. These findings highlight the need for enhanced SLE screening in young females with PSN.

Investigating the association between clinical manifestations—particularly initial symptoms—and PSN is essential for developing SLE screening protocols in PSN populations. Current evidence indicates that PSN, skin rash, joint symptoms, fever, and alopecia are among the most frequent initial symptoms in SLE patients. PSN-SLE patients had distinct clinical manifestations: higher rates of rash, alopecia, arthritis, and Raynaud syndrome. Raynaud syndrome, a vascular manifestation of SLE, may reflect shared pathogenic mechanisms (e.g., endothelial dysfunction) with PSN. In contrast, non-PSN-SLE patients more often presented with renal or hematological damage, leading to higher misdiagnosis rates (64.57% vs. 44.56%) [22–24]. Our findings align with these reports. Notably, skin rash—one of the most prevalent SLE manifestations—demonstrates a strong correlation with PSN, and rash, as a visible specific SLE sign, improved initial diagnosis accuracy (55.45% vs. 35.43%) [25]. Our study revealed a significantly higher incidence of rash as both initial and presenting symptom in PSN-SLE patients compared to non-PSN-SLE counterparts. Furthermore, PSN patients with rash exhibited substantially lower misdiagnosis rates than those without rash. Beyond rash, we observed increased prevalence of Raynaud syndrome as initial and presenting symptom in PSN-SLE patients. Our findings confirmed that PSN-SLE patients demonstrate significantly higher frequencies of rash, alopecia, arthritis, and Raynaud syndrome. Correlation analysis identified these as primary factors associated with PSN, indicating their close relationship. Given the visibility and diagnostic specificity of these SLE-directed signs, these four clinical features warrant inclusion as screening indicators in subsequent SLE investigations in PSN individuals.

Immunological indicators are fundamental to SLE classification criteria. The widely accepted paradigm posits that interactions among genetic, environmental, and immunological factors drive production and modulation of circulating autoantibodies—including ANA, anti-Sm, anti-SSA, and anti-dsDNA antibodies—culminating in target organ damage. Retrospective data from two SLE cohort studies demonstrate that autoantibodies may precede clinical symptoms by substantial periods [26, 27]. Specifically, one study of stored serum from routine US military health evaluations identified ANA in 88% (114/130) of service members subsequently diagnosed with SLE, while the other study of specimens from European medical biobanks and obstetric serum repositories identified ANA in 63% (22/35) of individuals later confirmed with SLE. These findings underscore ANA’s significant predictive value for SLE, warranting its incorporation as a screening indicator in subsequent SLE screening research among PSN individuals. PSN-SLE patients had higher positive rates of anti-Sm, anti-SSA, and anti-SSB antibodies. Anti-SSA/SSB antibodies are known to correlate with PSN, as UV radiation induces keratinocyte apoptosis and SSA antigen release, triggering autoimmune responses [28, 29]. Decreased C3/C4 levels in PSN-SLE patients reflect complement activation by immune complexes, a key SLE pathogenic process. This association may involve SSA antigen release during apoptosis of keratinocytes in PSN-SLE patients, ultimately contributing to rash pathogenesis. Investigations of the relationship between anti-SSA/SSB antibodies and the clinical features of lupus nephritis have found that positivity for these antibodies indicates a poor prognosis [30], highlighting the predictive value of autoantibody profiles for organ damage in SLE patients. Given these relationships, anti-SSA antibody assessment is particularly crucial in PSN-SLE evaluation. Fredi et al. identified correlations between anti-Sm antibodies and PSN manifestations in SLE patients [31]. Research examining the laboratory and clinical items of newer SLE classification criteria demonstrates significant associations between specific cutaneous presentations (particularly PSN-associated malar rash, alopecia, and oral ulcers) and autoantibody profiles [32], and these profiles exhibit predictive value for organ damage in SLE patients [33]. A retrospective study of 467 rheumatoid factor (RF)-positive systemic lupus erythematosus (SLE) patients identified RF as a renal damage protective factor potentially mediated by elevated anti-SSA antibody levels; meanwhile, RF positivity—previously linked to SLE disease activity—may contribute to photosensitive lupus erythematosus (PSN) via immune-mediated skin damage, with Fedrigo et al. [34] reporting that such patients have distinct serological profiles associated with anti-SSA antibody expression, which may amplify UV-induced keratinocyte apoptosis and skin inflammation in PSN-SLE cases.

Literature reports suggest that PSN in SLE may be a clue to underlying complement deficiency, with clinical PSN frequently observed in SLE patients exhibiting C3 and/or C4 deficiencies [35], consistent with our finding that reductions in C3 and C4 levels occur more frequently among PSN-SLE patients compared to non-PSN individuals. Collectively, the distinct autoimmune antibody and complement abnormalities observed in PSN-SLE versus non-PSN-SLE patients suggest divergent pathogenic mechanisms, warranting targeted therapeutic approaches including photoprotection.

Currently, photoprotection represents the primary approach for preventing PSN. International studies investigating photoprotective behaviors among SLE patients have found higher photoprotection practice scores in females, patients with higher education levels, and those with internet access, and that photoprotection awareness and PSN manifestations are closely correlated with appropriate photoprotective practices [36]. Janthongsri et al. [37] examined photoprotection habits and influencing factors among pediatric SLE patients, revealing insufficient photoprotection in most children and an inverse correlation between disease duration and sun exposure, suggesting photoprotection influences disease progression. Hydroxychloroquine (HCQ) has become a fundamental medication for SLE, demonstrating established efficacy both as monotherapy and in combination regimens [38, 39]. Wang Fan et al. [40] conducted follow-up studies on hospitalized SLE patients stratified by HCQ usage, finding HCQ not only provides therapeutic benefits but also improves long-term prognosis and enhances survival rates. Frodlund et al. [39] similarly identified HCQ use as a protective factor against organ damage. Studies further indicate HCQ exerts protective effects in incomplete SLE patients by reducing organ damage, delaying SLE-related disease progression, and improving quality of life and survival rates [41]. Khellaf et al. [42] found that adding HCQ treatment for patients with thrombocytopenia and elevated ANA titers who did not meet definitive SLE criteria yielded favorable therapeutic outcomes, confirming HCQ’s safety profile. Implementing enhanced photoprotection during follow-up for patients with SLE predisposition or early-stage SLE, supplemented with HCQ or other medications when indicated, not only mitigates UV-induced skin lesions but also facilitates control over systemic disease progression. PSN serves as an early SLE marker. Routine screening of PSN patients for associated clinical (rash, alopecia, arthritis, Raynaud syndrome) and immunological (anti-Sm/SSA/SSB, decreased C3/C4, RF) markers may facilitate early diagnosis. Photoprotection (sun avoidance, UV-blocking sunscreen) is critical for PSN-SLE patients to prevent disease exacerbation [15–18]. While hydroxychloroquine is effective for SLE treatment [38–40], this study did not assess its impact on PSN, which warrants future investigation.

## Limitations

This study has limitations: single-center design, small sample size, and short follow-up duration. Selection bias may exist due to the retrospective nature. Future multi-center prospective studies with larger samples and long-term follow-up are needed to validate findings. Additionally, the impact of photoprotection and medications (e.g., hydroxychloroquine) on PSN outcomes should be explored.

## Conclusions

1. PSN is more prevalent in female and young (< 40 years) SLE patients, correlating with rash, alopecia, arthritis, Raynaud syndrome, anti-Sm/SSA/SSB antibodies, decreased C3/C4, and RF positivity.
2. PSN-SLE patients have higher initial diagnosis accuracy, while non-PSN-SLE patients are more prone to misdiagnosis due to atypical manifestations (renal/hematological damage).
3. Routine screening of PSN patients for the identified clinical and immunological markers may facilitate early SLE diagnosis and improve prognosis.

## Supplementary Information

Supplementary Material 1.

## Data Availability

The datasets used in this study are available from the corresponding author upon reasonable request.
