# Supplementary material for: A study of clinical manifestations and associated factors in photosensitive patients with systemic lupus erythematosus
Source: Eur J Med Res. 2026 Jan 6;31:210. doi: 10.1186/s40001-025-03795-7 (PMC12870496; doi:10.1186/s40001-025-03795-7)
Supplement: Supplementary file 1 — Supplementary Material 1. [file 40001_2025_3795_MOESM1_ESM.docx]

Supplementary Material 1: 1997 ACR Classification Criteria for Systemic Lupus Erythematosus (SLE)

| **Criterion Category** | **Specific Criteria Description** |
| --- | --- |
| Clinical Criteria 1: Malar Rash | Fixed erythema over the malar eminences, spares the nasolabial fold, may be flat or elevated. |
| Clinical Criteria 2: Discoid Rash | Erythematous, raised patches with adherent keratotic scaling and follicular plugging; may progress to atrophic scarring. |
| Clinical Criteria 3: Photosensitivity | Skin rash induced by exposure to ultraviolet (UV) radiation (patient-reported or physician-observed). |
| Clinical Criteria 4: Oral Ulcers | Painless oral or nasal mucosal ulcers (observed by a physician). |
| Clinical Criteria 5: Arthritis | Non-erosive arthritis involving ≥2 peripheral joints, characterized by pain, swelling, or effusion. |
| Clinical Criteria 6: Serositis | - Pleuritis: Pleuritic chest pain, pleural rub, or pleural effusion.  - Pericarditis: Pericardial pain, pericardial rub, or pericardial effusion (documented by echocardiography or radiography). |
| Clinical Criteria 7: Renal Damage | - Persistent proteinuria: ≥0.5 g/24 hours or ≥3+ on qualitative urine protein testing.  - Cellular casts: Red blood cell, granular, hemoglobin, or mixed casts in urine sediment. |
| Clinical Criteria 8: Neurological Involvement | - Seizures: Excluding those induced by metabolic abnormalities (e.g., uremia), infections, or drugs.  - Psychosis: Severe disturbance of reality (hallucinations, delusions, disorganized behavior) excluding uremia or drug-induced causes. |
| Clinical Criteria 9: Hematological Disorder | - Hemolytic anemia with reticulocytosis.  - Leukopenia: White blood cell count <4×10⁹/L on ≥2 occasions (excluding drug-induced causes).  - Lymphopenia: Lymphocyte count <1.5×10⁹/L on ≥2 occasions (excluding drug-induced causes).  - Thrombocytopenia: Platelet count <100×10⁹/L (excluding drug-induced bone marrow suppression). |
| Immunological Criteria 10 | - Positive anti-double-stranded DNA (dsDNA) antibody (above laboratory reference range).  - Positive anti-Sm antibody (specific for SLE).  - Positive antiphospholipid antibody (a) IgG/IgM anticardiolipin antibody (above 95th percentile), (b) positive lupus anticoagulant test, or (c) false-positive syphilis serology (TPI or FTA-ABS negative) for ≥6 months. |
| Immunological Criteria 11 | Positive antinuclear antibody (ANA) |

**Supplementary Material 2: Clinical SLEDAI-2000 score**

| **Score** | **Clinical/Laboratory Manifestation** | **Notes/Exclusions** |
| --- | --- | --- |
| 8 | Seizure | Exclude metabolic (e.g., electrolyte imbalance), infectious, or drug-induced causes. |
| 8 | Psychosis | Severe disturbance of reality (hallucinations, incoherence, catatonic behavior); exclude uremia or drug causes. |
| 8 | Organic Brain Syndrome | Impaired orientation/memory, +≥2 of: perceptual disturbance, incoherent speech, insomnia/daytime drowsiness; exclude uremia, infection, or drugs. |
| 8 | Visual Disturbance | SLE-related retinal changes (hemorrhages, serous exudates, optic neuritis); exclude hypertension, infection, or drugs. |
| 8 | Cranial Nerve Disorder | New-onset sensory/motor neuropathy involving cranial nerves (e.g., facial palsy). |
| 8 | Lupus Headache | Severe, persistent headache; non-responsive to narcotic analgesia. |
| 8 | Cerebrovascular Accident (CVA) | New-onset stroke; exclude arteriosclerosis or embolic causes unrelated to SLE. |
| 8 | Vasculitis | Periungual infarction, tender finger nodules, or biopsy/proven vasculitis; exclude other vasculitides. |
| 4 | Arthritis | ≥2 joints with pain + inflammatory signs (tenderness, swelling, effusion). |
| 4 | Myositis | Proximal muscle weakness + elevated CK/aldolase, abnormal EMG, or biopsy-proven myositis. |
| 4 | Urinary Casts | New-onset red blood cell, granular, or hemoglobin casts. |
| 4 | Hematuria | >5 red blood cells/high-power field (HPF); exclude urinary tract infection, stones, or trauma. |
| 4 | Proteinuria | >0.5 g/24 hours (quantitative) or ≥3+ (qualitative); exclude pre-existing renal disease. |
| 4 | Pyuria | >5 white blood cells/HPF; exclude urinary tract infection. |
| 2 | Alopecia | New-onset patchy or diffuse hair loss (non-iatrogenic). |
| 2 | Rash | New-onset inflammatory rash (e.g., malar rash, discoid rash, maculopapular rash). |
| 2 | Mucosal Ulcers | New-onset oral or nasal ulcers (painful or painless). |
| 2 | Pleuritis | Pleuritic chest pain + pleural rub/effusion (auscultation or imaging). |
| 2 | Pericarditis | Pericardial pain + rub/effusion (auscultation or echocardiography). |
| 2 | Low Complement | Decreased CH50, C3, or C4 (below laboratory reference range). |
| 2 | Increased Anti-dsDNA Antibody | Titer above laboratory reference range or >25% by Farr assay. |
| 1 | Fever | ≥38℃ (100.4℉); exclude infection or drug-induced fever. |
| 1 | Decreased Platelets | Platelet count below laboratory reference range; exclude drug-induced thrombocytopenia. |
| 1 | Decreased White Blood Cells (WBC) | WBC <3×10⁹/L; exclude drug-induced leukopenia. |

Note: ≥15 for severe activity; 5-14 for mild to moderate activity; 0-4 for essentially no activity.

Supplementary Material 3: SLICC Damage Index for Organ Damage Assessment in SLE

| **Organ System** | **Damage Item** | **Description** | **Scoring Note** |
| --- | --- | --- | --- |
| Neuropsychiatric | 1. Seizure Disorder | Recurrent seizures requiring lifelong anticonvulsant therapy. | 1 point |
|  | 2. Psychosis | Permanent cognitive impairment or psychotic disorder (e.g., schizophrenia-like illness) requiring ongoing treatment. | 1 point |
|  | 3. Organic Brain Syndrome | Permanent memory loss, dementia, or focal neurological deficits (e.g., aphasia, hemiplegia) from CVA or SLE-related neurotoxicity. | 1 point |
|  | 4. Cranial Nerve Palsy | Permanent sensory/motor deficit from cranial nerve damage (e.g., optic atrophy, facial paralysis). | 1 point |
|  | 5. Peripheral Neuropathy | Permanent distal sensory/motor neuropathy (e.g., mononeuritis multiplex) unresponsive to treatment. | 1 point |
| Renal | 1. Chronic Renal Failure | Estimated glomerular filtration rate (eGFR) <60 mL/min/1.73m² for ≥6 months, or end-stage renal disease (ESRD) requiring dialysis/transplant. | 1 point (ESRD = 2 points if transplant/dialysis-dependent) |
|  | 2. Proteinuria | Persistent proteinuria ≥1 g/24 hours for ≥6 months despite treatment (not due to active nephritis). | 1 point |
|  | 3. Hypertension | Treatment-dependent hypertension (≥140/90 mmHg) for ≥6 months, attributed to SLE renal damage. | 1 point |
| Musculoskeletal | 1.Avascular Necrosis (AVN) | Radiologically confirmed AVN of ≥1 joint (e.g., hip, knee) requiring surgery or chronic pain management. | 1 point per joint (max 2 points) |
|  | 2. Myopathy | Permanent proximal muscle weakness (e.g., difficulty climbing stairs) from chronic myositis. | 1 point |
|  | 3. Joint Deformity | Permanent joint contractures or deformities (e.g., swan-neck fingers) from chronic arthritis. | 1 point per joint group (max 2 points) |
| Cardiopulmonary | 1. Pericardial Fibrosis | Constrictive pericarditis or pericardial calcification (imaging-proven). | 1 point |
|  | 2. Pulmonary Fibrosis | Interstitial lung disease with permanent restrictive defect (FEV1/FVC <70%, imaging-proven fibrosis). | 1 point |
|  | 3. Pulmonary Hypertension | Mean pulmonary arterial pressure ≥25 mmHg at rest (right heart catheterization-proven) for ≥6 months. | 1 point |
|  | 4. Coronary Artery Disease | Myocardial infarction, angina pectoris, or coronary revascularization (attributed to SLE or its treatment, e.g., corticosteroids). | 1 point |
| Gastrointestinal | 1. Hepatic Damage | Chronic liver disease (e.g., cirrhosis, portal hypertension) with elevated liver enzymes for ≥6 months. | 1 point |
|  | 2. Pancreatitis | Recurrent pancreatitis requiring dietary restrictions or enzyme replacement. | 1 point |
|  | 3. Intestinal Obstruction | Chronic bowel obstruction from adhesions or vasculitis (requiring surgery). | 1 point |
| Hematological | 1. Splenectomy | Surgical splenectomy for SLE-related thrombocytopenia or hemolysis. | 1 point |
|  | 2. Persistent Anemia | Chronic anemia (hemoglobin <10 g/dL) requiring lifelong iron or erythropoietin therapy. | 1 point |
| Dermatological | 1. Scarring Alopecia | Permanent hair loss with scarring (e.g., discoid lupus sequelae). | 1 point |
|  | 2. Skin Atrophy | Widespread cutaneous atrophy or ulceration (non-healing for ≥6 months). | 1 point |
| Ophthalmological | 1. Cataracts | Steroid-induced cataracts requiring surgical removal. | 1 point |
|  | 2. Glaucoma | Steroid-induced glaucoma with permanent visual field loss. | 1 point |
|  | 3. Retinal Damage | Permanent retinal scarring or optic neuritis leading to visual impairment (e.g., blindness in 1 eye). | 1 point per eye (max 2 points) |

Renal Damage: Based on confirmed SLE diagnosis, the presence of clinical features or laboratory abnormalities including persistent/recurrent proteinuria , casts, or abnormal renal biopsy findings.

Neurological Damage: Based on confirmed SLE diagnosis, the presence of CNS manifestations accompanied by abnormal result in ≥1 item among cerebrospinal fluid analysis, EEG, cranial MRI, or CT, excluding infections, uremic encephalopathy, electrolyte imbalance, hypertensive encephalopathy, psychiatric disorders, hepatic encephalopathy, or hormonotherapy-induced mental disorders.

Hematological Damage: Based on confirmed SLE diagnosis, the presence of abnormalities in ≥1 system: WBC <4.0×10^9^/L, hemoglobin <110g/L, or PLT <100×10^9^/L.
